# The role of blood nitric oxide level in predicting return of spontaneous circulation: a prospective case-control study

**DOI:** 10.1590/1806-9282.20240155

**Published:** 2024-09-02

**Authors:** Atıf Bayramoğlu, Erdal Tekin, Engin Kurt, Kamber Kaşali, Nezahat Kurt

**Affiliations:** 1 Alanya Alaaddin Keykubat University, Faculty of Medical, Department of Emergency – Alanya, Turkey.; 2 Atatürk University, Faculty of Medicine, Department of Emergency Medicine – Erzurum, Turkey.; 3 Mengücek Gazi Training and Research Hospital – Erzincan, Turkey.; 4 Atatürk University, Faculty of Medicine, Department of Medical Statistics – Erzurum, Turkey.; 5 Erzincan Binali Yıldırım University, Faculty of Medicine, Department of Biochemistry – Erzincan, Turkey.

**Keywords:** Cardiopulmonary resuscitation, Nitric oxide, Rosc

## Abstract

**OBJECTIVE:**

The aim of this study was to investigate whether there is a difference in serum nitric oxide levels between patients who return spontaneously after cardiopulmonary resuscitation and those who do not. We also examined the potential of using serum nitric oxide levels as a marker to make an accurate decision about patient survival.

**METHODS:**

We included 100 consecutive patients who were brought to the emergency clinic due to cardiac arrest. Blood samples were taken from these patients at admission, 30 min after admission, and when resuscitation was terminated.

**RESULTS:**

We found that there was a significant difference in NO1 and NO3 values between the group of patients who did not return after cardiopulmonary resuscitation and the group in which spontaneous circulation returned. The NO1 value was significant in the receiver operating characteristic (ROC) analysis, while the NO3 value was not. A higher NO1 value provided a higher rate of survival.

**CONCLUSION:**

Our findings suggest that nitric oxide may be a useful parameter to support the decision about patient survival. A higher NO1 value is associated with a better prognosis and survival rate. Therefore, serum nitric oxide levels may be a suitable indicator to support the decision-making process regarding patient survival.

## INTRODUCTION

Cardiovascular diseases are the leading cause of death worldwide, with 17.9 million people every year^1-3^. In the past 50 years, the survival rates for sudden cardiac deaths have remained low despite the application of cardiopulmonary resuscitation (CPR), electrical defibrillation, and other advanced resuscitation techniques^4^. Resuscitations performed outside the hospital have a survival rate between 1 and 6%^5-7^, and those performed by emergency health services have a survival rate between 5 and 10%^7,8^. Ventricular fibrillation is the most common arrhythmia in adult patients who undergo cardiopulmonary arrest^9^.

Due to its enlargement, vasomotor tone, and active metabolic state that produces mediators for coagulation and inflammation, the endothelium is an effective area for damage repair in cases of ischemia-reperfusion injury. The endothelium conducts this repair function by releasing nitric oxide (NO), endothelin 1, and prostacyclins^10^. NO activates vasodilation, anti-inflammation, anti-apoptosis, and antioxidant effects and inactivates platelets and leukocytes^11^. Moreover, in cases of the overproduction of NO associated with various nervous system diseases, NO seems to become an important neurotoxin^12^.

On the other hand, when giving patients CPR, it is difficult to decide when to stop. There is less information in the literature to guide this decision^3-5^, with the most important data available concerning the level of the end-tidal carbon dioxide (EtCO_2_): an extremely low EtCO_2_ (<10 mmHg) after prolonged resuscitation (>20 min) is a sign of deficient circulation and a strong predictor of acute mortality^13,14^. Moreover, this decision—which the American Heart Association (AHA) emphasized in its Advanced Cardiac Life Support Guidelines, 2015—should not be solely based on the EtCO_2_ value, which may be considered only one among other parameters for terminating resuscitation^15^.

Therefore, considering the repairing function of NO and the uncertainty regarding when to terminate CPR, in this study, we hypothesize that the blood NO levels of those patients who got the return of spontaneous circulation (ROSC) after the standard resuscitation protocols were applied would be different from those who did not get the ROSC. In addition, we aimed to investigate the potential of quantitative serum NO as a marker and tool for the accurate determination of death.

## METHODS

### Study design

The study was conducted in accordance with the ethical principles stated in the Declaration of Helsinki. All participants or their relatives provided written informed consent before their inclusion in the study. This study was conducted according to a single-center prospective case-control design approved by the local ethics committee (Meeting no. 5, Decision no. 40/2019).

### Setting

The study was conducted in a tertiary emergency department (ED). The study was conducted prospectively between July 2019 and February 2021. Patients who had a heart attack in the ED, whether brought in by ambulance or by their relatives, and patients who developed a heart attack during their follow-up in the ED were included.

Blood samples were taken from these patients (NO1), and resuscitation was performed according to the AHA 2015 Advanced Life Support Guidelines. Blood samples were taken again when ROSC was provided or when the decision of death was made (NO3). In patients whose resuscitation took longer than 30 min, one more blood sample was taken at the 30th minute, resulting in a total of three blood samples taken from these patients (NO2).

### Participants

Patients aged 18 years and over who underwent resuscitation (defined as cessation of heartbeat and breathing) in the emergency department were included in the study. Patients with trauma, intracerebral lesion, brain hematoma, cerebral hemorrhage, intracranial tumors, terminal malignancy, end-stage lung fibrosis, and chronic systolic heart failure with an ejection fraction less than 20% were excluded.

The study included 100 consecutive patients who underwent resuscitation in the emergency department. Patients not included in the study are: 21 patients with trauma, 4 with intracerebral lesion, 7 with cerebral hemorrhage, 15 with terminal malignancy, and 9 patients with chronic systolic heart failure with an ejection fraction less than 20%.

### Variables

The serum NO levels of the patients were analyzed using the Nitrate/Nitrite Colorimetric Assay Kit and the spectrophotometric method. The coefficient of variation of 84 samples was 2.7%, and that of five samples was 3.4%, according to the package insert of the kit.

### Measurements

From each patient, a 5 mL blood sample was taken in a gel biochemistry blood tube. The blood samples were collected in a centrifuge at 3,500 rpm for 10 min. After centrifugation, the serum phase at the top of the tube was transferred to Eppendorf tubes by aliquoting. All the samples were stored in a deep freezer at -80°C until the day of NO analysis. In March 2021, all the serum samples were thawed at +4°C, and the NO levels were measured using the Nitrate/Nitrite Colorimetric Assay Kit.

### Statistical analysis

Data were presented as mean, standard deviation, median, minimum, maximum, percentage, and number. The normal distribution of quantitative variables was evaluated using the Shapiro-Wilk W-test. The Pearson chi-square test was used to compare categorical variables. Independent sample t-tests were used to compare quantitative variables between the survivors and deceased groups when the normal distribution condition was met, and the Mann-Whitney U test was used when it was not. The repeated measures ANOVA test was used to compare the quantitative variables of more than two dependent groups when the condition of normal distribution was met, and the Friedman test was used when it was not.

The predictive estimators were investigated using receiver operating characteristic (ROC) analysis to determine whether the quantitative variables of the survivors and deceased had distinguishing features. The validity of diagnostic test results was checked by calculating sensitivity, specificity, positive predictive value, negative predictive value, prevalence, positive likelihood ratio, negative likelihood ratio, and accuracy. The power of the variable to distinguish between the deceased and survivors was also determined. All analyses were performed using the IBM SPSS 22 version. A statistical significance level of p<0.05 was accepted.

### Sample size calculation

The sample size calculation was based on a previous study^16^ that reported the mean NO value of non-survivors and survivors as 42.53 μmol/L and 77.09 μmol/L, respectively. With a power of 80% and an alpha level of 0.05, a total of 100 patients (50 in each group) were needed to detect a significant difference between the two groups.

## RESULTS

A total of 100 patients were included in the study, with 64% (64) being male and a mean age of 69±13 years (minimum 32, maximum 91). The demographic data are summarized in Table 1.

**Table 1:** Demographic data of the study and analysis of categorical variables affecting prognosis among groups.

Demographic data		Survivor (n=40)	Deceased (n=60)	p-value	Total
Age mean±SD (min–max)		65.52±15.1 (32–91)	71.13±11.33 (42–88)	0.037	68.89±13.2 (32–91)
Gender (male)%		65% (26)	63.3% (38)	0.87	64% (64)
Compression (mechanical)		50% (30)	35% (14)	0.14	44% (44)
Initial rhythm		Asystole 60% (24) VF 30% (12) PEA 10% (4)	Asystole 85% (51) VF 1.7% (1) PEA 13.3% (8)	<0.001	Asystole 74.7% VF 13.1% PEA 12.1%
Place		OHCA 70% (28) İHCA 30% (12)	OHCA 80% (48) İHCA 20% (12)	0.25	OHCA 75.8% İHCA 24.2%
Variables		Survivors/(survivors+deceased) %		Chi-square	p-value
Rhythm	VF	12/13 (92.3%)		17.043	<0.001
	PEA	4/12 (33.3%)			
	Asystole	24/75 (32%)			
Place	OHCA	28/76 (36.8%)		1.316	0.251
	İHCA	12/24 (50%)			
Compression	Mechanical	14/44 (31.8%)		2.192	0.139
	Manual	26/56 (46.4%)			
Gender	Male	26/64 (40.6%)		0.029	0.865
	Female	14/36 (38.9%)			

SD: standard deviation; VF: ventricular fibrillation; PEA: pulseless electrical activity; OHCA: out-of-hospital cardiac arrest; IHCA: in-hospital cardiac arrest.

The relationship between categorical variables and prognosis was analyzed and presented in Table 1. There was a significant relationship between the patients’ arrival rhythm and prognosis, but no significant relationship was found between the location of the arrest, the manual or mechanical application of compression, and the gender of the patient and prognosis.

The NO1 and NO3 data were normally distributed, while the NO2 data were not. The results in Table 2 show a significant correlation between prognosis and the NO1 and NO3 variables (p<0.05).

**Table 2:** Comparison of nitric oxide levels.

Prognosis	Deceased					Survivor				t, Z		p
	Valid, n	Mean±SD		Median (min–max)		Valid, n	Mean±SD	Median (min–max)				
NO1	59	12.77±8.52		10.69 (1.74–52.33)		39	21.68±14.47	18.91 (2.32–70.65)		3.470		0.001
NO2	26	12.52±7.43		11.97 (2.89–30.1)		8	19.9±13.44	15.95 (8.15–50.03)		1.584		0.113
NO3	56	12.22±7.11		9.94 (2.32–30.57)		39	16.78±11.79	13.73 (0.76–44.86)		2.157		0.035
Cutoff value for NO1	Sensitivity		Specificity		Positive predictive value	Negative predictive value	Positive likelihood ratio		Negative likelihood ratio		Accuracy ratio	
14.96	0.68		0.74		0.80	0.60	2.64		0.43		0.70	
16.97	0.78		0.67		0.78	0.67	2.34		0.33		0.73	
18.12	0.81		0.59		0.75	0.68	1.98		0.32		0.72	

**Figure 1:**
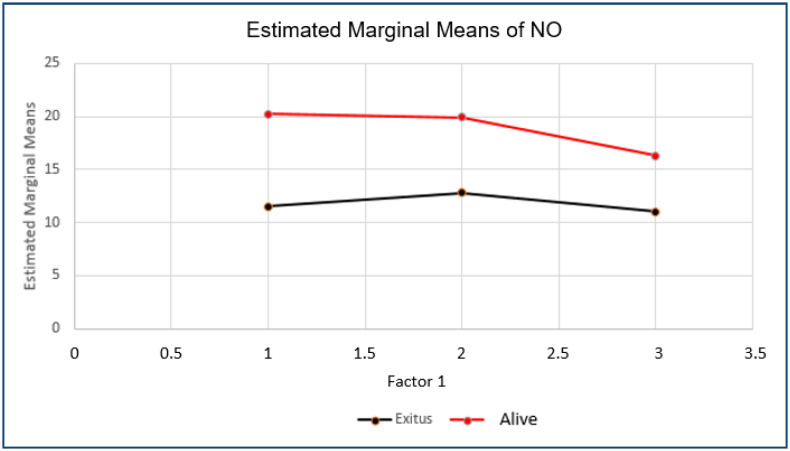
Averages of NO1, NO2, and NO3 values.

The change in NO values over time (initial, second, and last measurements) for survivors and deceased are compared and given in Figure 1. No significant difference was found between the changes in NO values over time in both groups.

ROC analysis was used to investigate whether NO1 and NO3 variables could be used in estimation. While NO1 was found to be statistically significant (p=0.001), NO3 was not (p=0.163). The NO1 cutoff value was determined using the Youden index, and the calculated value was 16.9754. Table 2 presents the sensitivity, specificity, positive predictive value, negative predictive value, positive likelihood ratio, negative likelihood ratio, and accuracy ratio for the validity of the diagnostic test results for the two cutoff values with a high Youden index.

## DISCUSSION

In our study, the NO1 value was observed to be significantly different in patients who died after CPR and those with ROSC. This confirms the hypothesis that there would be a difference between the blood NO quantitative levels of patients with ROSC and those without. Although results supporting our finding are available in animal studies^11,17^, this is the first time this difference has been demonstrated in a study on humans. This study also demonstrates that ROSC is more likely in patients with a high NO1 value. Moreover, a significant difference was found between the two groups in terms of their NO3 values, reinforcing the idea that there is a significant difference between the exitus patient group and the group with ROSC and that NO level is an effective factor in determining whether to end resuscitation. In contrast, no significant difference in NO2 values was found between the deceased group and the group with ROSC, suggesting that patients with high NO1 values might have ROSC in less than 30 min at a high rate and that patients whose resuscitation lasted longer than 30 min might generally have a low initial NO1 value. While only eight patients with low NO1 values survived, 26 of them died, indicating that there may not be a significant difference between the groups in terms of the NO2 value and that NO is an effective factor for ROSC but not the only one. In future studies on this subject, determinative factors other than NO should be considered.

ROC analysis was used to examine the potential of the NO1 and NO3 values as markers for supporting the death decision, and the NO1 value was found to be statistically significant (p=0.001) unlike the NO3 value (p=0.163). The NO value's sensitivity and specificity for the cutoff value of 16.97 were 78 and 67%, respectively. The accuracy rate was determined to be 73%. Also, when we accepted the cutoff value as 18.12, the patients who would survive were indicated with an accuracy of 81%. Moreover, when we accepted the cutoff value as 14.96, the specificity rate became 74%. Thus, consistent with the results in animal studies, it was found that the NO value has the potential to be a parameter to support the death decision in humans^11,17,18^.

In conclusion, the NO value at the time of cardiopulmonary arrest is an important factor for ROSC; patients with a high baseline NO seem to have a significantly higher chance of ROSC. However, this study is limited: its results cannot be generalized as it had only 100 participants and was conducted at a single center. Therefore, more detailed and multi-centered studies that focus on subgroup analyses—such as patients’ admission rhythm and age range—may provide more useful and generalizable results. However, nitric oxide levels may be different depending on the initial rhythm and age of the patient. These changes may affect the interventions that need to be made.
